# Validation of the person-centered maternity care scale at governmental health facilities in Cambodia

**DOI:** 10.1371/journal.pone.0288051

**Published:** 2023-07-06

**Authors:** Yuko Takahashi Naito, Rieko Fukuzawa, Togoobaatar Ganchimeg, Patience A. Afulani, Hirotsugu Aiga, Rattana Kim, Asako Takekuma Katsumata

**Affiliations:** 1 Graduate School of Comprehensive Human Sciences, University of Tsukuba, Tsukuba, Japan; 2 Faculty of Medicine, University of Tsukuba, Ibaraki, Japan; 3 Departments of Epidemiology & Biostatistics and Obstetrics, Gynecology, & Reproductive Sciences, University of California, San Francisco (UCSF), San Francisco, CA, United States of America; 4 School of Tropical Medicine and Global Health, Nagasaki University, Nagasaki, Japan; 5 National Maternal and Child Health Center, Phnom Penh, Cambodia; Arba Minch University, ETHIOPIA

## Abstract

**Background:**

Women’s childbirth experience of interpersonal care is a significant aspect of quality of care. Due to the lack of a reliable Cambodian version of a measurement tool to assess person-centered maternity care, the present study aimed to adapt the “Person-Centered Maternity Care (PCMC) scale” to the Cambodian context and further determine its psychometric properties.

**Methods:**

The PCMC scale was translated into Khmer using the team translation approach. The Khmer version of PCMC (Kh-PCMC) scale was pretested among 20 Cambodian postpartum women using cognitive interviewing. Subsequently, the Kh-PCMC scale was administered in a survey with 300 Cambodian postpartum women at two governmental health facilities. According to the COnsensus-based Standards for the Selection of health status Measurement Instruments (COSMIN) standard, we performed psychometric analysis, including content validity, construct validity, criterion validity, cross-cultural validity, and internal consistency.

**Results:**

The preliminary processes of Kh-PCMC scale development including cognitive interviewing and expert review ensured appropriate levels of content validity and acceptable levels of cross-cultural validity of the Kh-PCMC scale with four-point frequency responses. The Scale-level Content Validity Index, Average (S-CVI/Avg) of 30-item Kh-PCMC scale was 0.96. Twenty items, however, performed optimally in the psychometric analysis from the data in Cambodia. The 20-item Kh-PCMC scale produced Cronbach’s alpha of 0.86 for the full scale and 0.76–0.91 for the subscales, indicating adequately high internal consistency. Hypothesis testing found positive correlations between the 20-item Kh-PCMC scale and reference measures, which implies acceptable criterion validity.

**Conclusions:**

The present study produced the Kh-PCMC scale that enables women’s childbirth experiences to be quantitatively measured. The Kh-PCMC scale can identify intrapartum needs from women’s perspectives for quality improvement in Cambodia. However, dynamic changes in and diverse differences of cultural context over time across provinces in Cambodia require the Kh-PCMC scale to be regularly reexamined and, when needed, to be further adjusted.

## Background

Person-centered maternity care is highlighted in the WHO Quality of Care Framework for Maternal and Newborn Health [1] and the WHO recommendations on intrapartum care for women’s positive childbirth experience [2]. Women’s childbirth experience is a vital indicator to measure both immediate and long-term outcome of quality of care [3–7].

Cambodia is one of nine successful countries in achieving the Millennium Development Goal (MDG) 5A of at least 75% reduction in their Maternal Mortality Ratio (MMR), from 1020 to 161 per 100,000 births between 1990 and 2015 [8]. However, several qualitative studies revealed that Cambodian women did not always receive women-centered care during childbirth at the health facility. Women’s poor perception on interpersonal elements of quality of care has been reported as a significant barrier to maternal health service utilization [9–12]. Not using facility health services lead delayed care seeking behavior and thereby delays in accessing life-saving medical interventions [13]. It further leads to significant losses in achieving Sustainable Development Goal (SDG) target 3.1 reduction in the global MMR to less than 70 per 100,000 live births by 2030 [14]. Despite general recognition of the importance of the quality of care, improvement of women’s childbirth experiences has not been adequately addressed despite the tremendous efforts made for increasing the quality of maternity care [15]. In Cambodia, very little is known about the current situation of women’s childbirth experiences, because there is limited evidence due to the absence of reliable tools for measuring them.

There are various tools to measure women’s childbirth experiences, but there is a lack of consensus in how to operationalize the constructs of person-centered maternity care. To date, the Person-Centered Maternity Care (PCMC) Scale is the only one validated tool that comprehensively covers the dimensions of the WHO Quality of Care Framework as process indicators, and which is based on standardized procedures for scale development including cognitive interviewing and psychometric analysis. The PCMC scale is a validated tool to measure women’s experiences of received maternity care during childbirth in facility settings in developing countries [16]. It was initially developed in Kenya and subsequently validated in India [17], and was used in Ghana [18]. Recently, a short version of PCMC scale was proposed using a data-driven approach, for application across multiple settings [19].

In general, cross-cultural research is required to address the situations where underlying concepts of key topics may not be identical or even comparable across cultures. Thus, a tool appropriate in one context may not be adequate in other contexts [20]. However, few earlier studies not only adequately pretested tools and reported how cultural contexts influence the tool adaptation and validation processes and how the challenges in those processes could be addressed [21]. A poorly translated and adapted tool is likely to lack equivalence to its original tool, thereby end up having inadequate validity, reliability and comparability [22].

This study, therefore, aimed to develop a reliable and valid PCMC scale in Khmer language for use in Cambodian postpartum women adapted from the validated PCMC scale from Kenya and India, considering the influence of cultural context.

## Materials and method

The present study followed the standard procedures of scale development recommended by DeVellis [23]: including translations; expert reviews; cognitive interviewing and pretests; and a survey for psychometric assessment. The study consisted of two phases. Phase 1 involved cross-cultural translation and adaptation of the Cambodian version of the scale using a qualitative approach. Phase 2 was a survey for psychometric assessment using a quantitative approach.

The study took place at two governmental health facilities in Cambodia. One urban hospital in Phnom Penh, national capital and one rural health center in Kampong Chhnang province were selected. The urban hospital provides both routine and emergency services including surgery and blood transfusion. Mean number of delivery cases at the hospital was reported as approximately 600 per month, of which 30% are caesarian deliveries. It serves a variety of clients (e.g. various domicile, diverse economic levels, different religions, and both low and high-risk pregnancies). The rural health center provides antenatal care, normal delivery, immunization, health education, and referral services. Its mean number of normal deliveries was approximately 10 per month, and there is no caesarian delivery. Note that forceps delivery is not in the clinical protocol in Cambodia.

The early postpartum women who delivered at the two facilities were recruited at the maternity ward before discharge. Women satisfying all the following eligibility criteria were included in the study: (i) aged 18–49 years; (ii) willing to participate; (iii) delivered at the target health facility; (iv) had a live birth; (v) able to understand the Khmer language. Women satisfying any of the following conditions were excluded from the study respondents: (i) not willing to participate in the study; (ii) had a stillbirth; (iii) having their infant hospitalized due to serious complications such as congenital diseases and cerebral palsy; and (iv) admitted for reasons other than childbirth.

### Phase 1: Translation of the PCMC scale and pretest

First, we obtained the permission to use the PCMC scale from the developer of the original PCMC scale, for the purpose of its adaptation to Cambodian contexts. A total of 31 items were used as initial item pool, i.e., all the original 30 items validated in Kenya [16] and one additional item ‘being asked to pay bribe’ validated in India [17]. The overall Phase 1 procedures were performed, by following the WHO guideline on translation and adaptation of instruments [24]. Important considerations for cross-cultural research are conceptual and cross-cultural equivalence.

The English version of the original PCMC scale items were first translated into Khmer language, employing the team translation approach. The translation team was composed of one bilingual English-Khmer translator, one bilingual Japanese-Khmer translator and two trilingual English-Khmer-Japanese linguistic experts. It was subsequently reviewed by eight Cambodian content experts to identify unnatural expressions and to review cultural appropriateness for the Cambodian context. The Khmer version of the PCMC (Kh-PCMC) scale was then pre-tested among 20 Cambodian postpartum women using cognitive interviewing from 20 January to 28 March 2021. We spent a substantial amount of time attempting to accurately capture the cultural context of Cambodia and select appropriate words and phrases. Discrepancies and nuanced translations were discussed and resolved among the translation team. The translation team and the tool developer approved the retention of the 31 items for use in a field survey. The details available in a separate paper [25].

### Phase 2: A survey for psychometric assessment

#### Data collection

Face-to-face interviews were conducted in Khmer from 4 April to 27 August 2021. The 31-items Kh-PCMC scale was administrated along with questions on socio-demographics, maternal characteristics, and outcome measures (satisfaction, future intention to deliver the same hospital). The data collectors read aloud each item and answer options, giving explanations and rephrasing when necessary, and allowed the women select the response that fits best from the answer options. The data collectors inputted the respondent’s answers to the online questionnaire using a smartphone or tablet computer, and data were uploaded directly to the cloud. To prevent missing data, we set up the online questionnaire so that it was only possible to proceed to the next section when all answers were entered. A total of 300 women were interviewed to achieve a minimum of five to ten subjects to one item recommended for exploratory factor analysis [23].

#### Psychometric analysis

The psychometric properties of the Kh-PCMC scale were assessed according to the COnsensus-based Standards for the Selection of health status Measurement Instruments (COSMIN) standards of Risk of bias checklist [26]. In this study, the five measurement properties of content validity, structural validity, internal consistency, criterion validity, and cross‐cultural validity, for the Kh-PCMC scale were assessed. Statistical analysis was performed using IBM SPSS version 27.

#### Data quality

Firstly, the normality of data distribution was determined using a one-sample Kolmogorov-Smirnov test (significance < 0.05) for descriptive variables. Univariate analysis was performed to determine the distribution of all the items. Where questions had a response option in the “not applicable” category, “not applicable” was recoded to the highest response category to obtain a uniform scale for the psychometric properties as described elsewhere [16]. Negative items were reverse coded to reflect a scale of 0 as the lowest level to 3 as the highest level.

The mean and standard deviation of each item were examined to assess floor and ceiling effects. As an initial examination of item performance, a correlation matrix was constructed.

(1) Content validity

Content validity, which refers to “the degree to which the content of a health-related patients-reported outcomes (HR-PRO) instrument is an adequate reflection of the construct to be measured” [27], is considered to be the most important measurement property [28]. Content validity is evaluated by subjective judgment from patients and professionals. The content validity index (CVI) [29,30] of the 31-items Kh-PCMC scale was assessed by eight Cambodian experts. The experts included a medical doctor, four midwives, an academic expert from nursing science with experience in instrument development, a WHO officer, and a government official. Two of eight content experts were monolinguals. The CVI of each item (I-CVI) was calculated as the ratio of the number of ‘3 = relevant with needs minor revisions’ and ‘4 = very relevant’ responses to the number of experts with 0.78 or above being preferred [28]. The overall CVI of the scale was calculated as the averaging calculation method (S-CVI/Ave) with 0.9 or above as the preferred outcome [29].

(2) Structural validity

Structural validity refers to “the degree to which the scores of an HR-PRO instrument are an adequate reflection of the dimensionality of the construct to be measured” [27]. The Kaiser-Meyer-Olkin (KMO) measure of sampling adequacy was examined to check the suitability of data for factor analysis. A KMO value of 0.5 or above is considered satisfactory as the criterion for sampling adequacy [31,32]. The initial exploratory factor analysis was performed to determine the number of factors to be retained by examining a scree plot of eigenvalues for all the 31 items. Both the Kaiser’s rule with eigenvalues greater than one [33] and the “break” in the scree plot [23] was used to determine the number of factors to extract, along with theoretical considerations. Multiple rounds of subsequent exploratory factor analysis were performed to examine the item loadings to determine which items to retain or delete. The acceptable factor loading was set to greater than 0.3 [34], while a lenient cut-off point of 0.1 was used to retain items in the India validation [17]. Factor rotations were applied to simplify the interoperability of factor solutions [35]. In the present study, Promax rotation was used to allow for correlations between the rotated factors. The use of Promax rotation was justified because the PCMC domains are theoretically correlated. We compared our factor structure to that obtained in Kenya validation and tested with confirmatory factor analysis.

(3) Internal consistency

The internal consistency reliability (homogeneity), which refers to “the degree of the interrelatedness among the items” [27], was assessed using Cronbach’s coefficient alpha. Cronbach’s alphas of 0.7 or higher are generally considered sufficient evidence of reliability for a new scale [36], or 0.8 or higher for a mature scale [23].

(4) Criterion validity

Criterion validity, which refers to “the degree to which the scores of an HR-PRO instrument are an adequate reflection of a gold standard” [27]. We employed hypothesis testing where gold standards are not available, which is assessed with Pearson correlation coefficients using P values (r,p) [26]. According to previous studies, we set hypotheses about the expected magnitude and direction of relationships between the Kh-PCMC scale and reference measures: satisfaction with care [4,7,37,38], quality of care rating [16], and the future intention to seek delivery care in the same facility if she were to be pregnant again [5,37]. Correlation coefficients under 0.3, between 0.3 and 0.6 and over 0.6, were considered low, moderate and high, respectively [39].

(5) Cross‐cultural validity

Cross‐cultural validity refers to “the degree to which the performance of the items on a translated or culturally adapted HR-PRO instrument are an adequate reflection of the performance of the items of the original version of the HR-PRO instrument” [27]. In this study, cross‐cultural validity is assessed according to cultural translation and adaptation process using team translation, expert reviews, and cognitive interviewing in phase 1.

### Ethics

The study received ethical approval form the Ethics Committee, Faculty of Medicine, University of Tsukuba on 24 December, 2020: Reference: IRB1605, and National Ethics Committee for Health Research, Ministry of Health Cambodia on 30 December, 2020: Reference: #322 NECHR. Oral informed consents were obtained from all respondents prior to participation. Participants were informed their participation in the study was voluntary, and they had the right to refuse to participate in the study and could withdraw at any time after giving their consent without giving any reasons. Interviews were conducted in a private space of the facilities. Participants’ responses were kept confidential by de-identifying the data using a unique identifier code.

## Results

### Respondent characteristics

A total of 300 postpartum Cambodian women were interviewed. Table 1 shows the demographic characteristics of respondents. The mean age of the women was about 29 years (range of 18 to 46) with the mean parity of 2.26 (range of 1 to 8 children). Almost all of the women were married (99.3%) and Buddhist (97.3%). About half (48.5%) of women had less than primary education, and 69.1% had some difficulty in reading the Khmer language or were illiterate; 6.6% were certified as the poorest to be exempt from paying medical expenses. The postpartum length for women interviewed was between one and seven days.

**Table 1:** Characteristics of 300 women.

Characteristics			Number	Percent
**Age (years)**				
	Mean (SD)		29.32	5.94
	< 20		13	4.3
	20–24		50	16.7
	25–29		94	31.3
	30–34		77	25.6
	35–39		53	17.7
	40 <		13	4.2
**Parity**				
	Mean (SD)		2.26	1.20
	1		89	29.6
	2		112	37.2
	3		54	17.9
	4		28	9.3
	5		15	5
	7<		2	0.6
**Marital status**				
	Married		299	99.3
	Widowed		1	0.3
**Religion**				
	Buddhism		293	97.3
	Khmer Muslim		6	2
	Cristian		1	0.3
**Occupation**				
	Housewife		125	41.5
	Factory worker		98	32.6
	Self-employed retail		36	12
	Company employee		18	6
	Farmer		15	5
	Government official		4	1.3
	Scavenger		4	1.3
**Education**				
	No		26	8.6
	Primary school		120	39.9
	Secondary school		95	31.6
	High school		48	15.9
	University		11	3.7
**Literacy**				
	Illiterate		55	18.3
	With some difficulty		153	50.8
	Very well		92	30.6
**Economical background**				
	Non-ID poor		280	93.4
	ID poor holder (the poorest)		20	6.6
**Postpartum day**				
	Mean (SD)		2.52	1.42
**Mode of delivery**				
		Vaginal delivery (normal)	196	65.1
		Vaginal delivery (episiotomy)	42	14
		Caesarean delivery	62	20.6

### Data quality

All data were normally distributed. While seven items (#7,21,22,23,29,30,31) were of a particularly high mean (+1SD) of greater than 2.9 in upper limit of 3, we retained all items at this stage (S1 Table).

### Score distribution of 30-item scale

The mean Kh-PCMC full score for the sample based on the sum of the original 30 items was 69.52 (SD = 9.47) with a range of 48 to 89 (where 0 is the worst score and 90 is the best score). The mean Kh-PCMC sub-scale scores for the sample were 16.01 (SD = 1.53) with a range of 8 to 18, 15.43 (SD = 3.92) with a range of 6 to 24, and 36.26 (SD = 4.38) with a range of 24 to 44, for dignity and respect, communication and autonomy, and supportive care, respectively. Standardized scores ranging from 0 to 100 are 77.02 for full score, and 88.94, 57.15, and 80.58 in three subscales, respectively. The score distribution and the distributions of 30-item Kh-PCMC scale are shown in S2 and S3 Tables, respectively.

### Psychometric properties

(1) Content validity

Table 2 shows Content Validity Index (CVI) evaluation of the 31 items by eight content experts. The S-CVI/Avg (scale-level content validity index, average) was 0.96 and the S-CVI/UA (scale-level content validity index, universal agreement) was 0.74 with a total item agreement of 23 of 31 items (7 items at 0.87, and 1 item at 0.75).

**Table 2:** CVI evaluation on a 31-item scale by eight experts.

Item		Expert 1	Expert 2	Expert 3	Expert 4	Expert 5	Expert 6	Expert 7	Expert 8	Number of agreement	Item CVI^1^
1	Time to care	✓	✓	✓	✓	✓	✓	✓	✓	8	1.00
2	Introduce self	-	-	✓	✓	✓	✓	✓	✓	6	0.75
3	Called by name	-	✓	✓	✓	✓	✓	✓	✓	7	0.87
4	Treated with respect	✓	✓	✓	✓	✓	✓	✓	✓	8	1.00
5	Friendly	✓	✓	✓	✓	✓	✓	✓	✓	8	1.00
6	Visual privacy	✓	✓	✓	✓	✓	✓	✓	✓	8	1.00
7	Record confidentiality	✓	✓	✓	✓	✓	✓	✓	✓	8	1.00
8	Involvement in care	✓	✓	✓	✓	✓	✓	✓	✓	8	1.00
9	Consent to procedures	✓	✓	✓	✓	✓	✓	✓	✓	8	1.00
10	Delivery position choice	✓	✓	✓	✓	✓	✓	✓	✓	8	1.00
11	Language	✓	✓	✓	✓	✓	✓	✓	✓	8	1.00
12	Explain exams/procedures	✓	✓	✓	✓	✓	✓	✓	✓	8	1.00
13	Explain medicines	✓	✓	✓	✓	✓	✓	✓	✓	8	1.00
14	Talk about feeling	✓	✓	✓	✓	✓	✓	✓	✓	8	1.00
15	Support anxiety	✓	✓	✓	✓	✓	✓	✓	✓	8	1.00
16	Able to ask questions	✓	✓	✓	✓	✓	✓	✓	✓	8	1.00
17	Labor support	✓	✓	✓	✓	✓	✓	✓	✓	8	1.00
18	Delivery support	✓	✓	✓	✓	✓	✓	✓	✓	8	1.00
19	Attention when need help	✓	✓	✓	✓	✓	✓	✓	✓	8	1.00
20	Control pain	✓	✓	✓	✓	✓	✓	✓	✓	8	1.00
21	Verbal abuse	✓	-	✓	✓	✓	✓	✓	✓	7	0.87
22	Physical abuse	✓	-	✓	✓	✓	✓	✓	✓	7	0.87
23	Bribes	✓	-	✓	✓	✓	✓	✓	✓	7	0.87
24	Enough staff	✓	✓	✓	✓	✓	✓	✓	✓	8	1.00
25	Took best care	✓	✓	✓	✓	✓	✓	✓	✓	8	1.00
26	Trust	✓	✓	✓	✓	✓	✓	✓	✓	8	1.00
27	Crowded	✓	-	✓	✓	✓	✓	✓	✓	7	0.87
28	Clean	✓	✓	✓	✓	✓	✓	✓	✓	8	1.00
29	Electricity	✓	-	✓	✓	✓	✓	✓	✓	7	0.87
30	Water	✓	-	✓	✓	✓	✓	✓	✓	7	0.87
31	Safe	✓	✓	✓	✓	✓	✓	✓	✓	8	1.00
										S-CVI/Ave^2^ =	0.96
										S-CVI/UA^3^ =	0.74
Number of agreements		29	24	31	31	31	31	31	31	Average proportion of agreement across experts⁴	0.96
Proportion of relevant		0.93	0.77	1.00	1.00	1.00	1.00	1.00	1.00		

^1^Item CVI = Number of experts rating the item either 3 or 4/total number of experts.

^2^S-CVI/Ave = Sum of the I-CVIs (I-CVI1+I-CVI2+I-CVI3+ ……. +I-CVIn)/total number of items. Averaging method.

^3^S-CVI/UA = Number of items that achieved rating 3 or 4 by all experts/total number of items. Universal agreement method.

⁴Average proportion of agreement across experts = Proportion of agreement of each expert/total number of experts.

"-" l = not relevant, 2 = unable to assess relevant, "✓" 3 = relevant with needs minor revisions, 4 = very relevant.

(2) Structural validity

The KMO values of 0.83 and the Bartlett’s test of sphericity (Chi-squared value = 3484.092 and df 465, *P* < 0.001) indicated that the overall variables were satisfactory for factor analysis.

The initial exploratory factor analysis using principal factor with 31 items yielded ten factors with one dominant factor with eigenvalues of greater than one, (7.22, 2.284, 2.077, 1.75, 1.624,) respectively, accounting for 66.83% of the total variance. Because the original PCMC scale has a three-factor structure, the second exploratory factor analysis was performed using principal factor and Promax rotation assuming a three-factor structure. The second exploratory factor analysis with 31 items yielded three factors including one dominant factor, 17 items loaded on the first factor, 11 on the second factor, and three on the third factor. If we used a cut-off of 0.3, 11 items would be eliminated, leaving 20 items. While if we used a cut-off of 0.1, two items (physical abuse and verbal abuse) would be eliminated, leaving 29 items.

**Fig 1:**
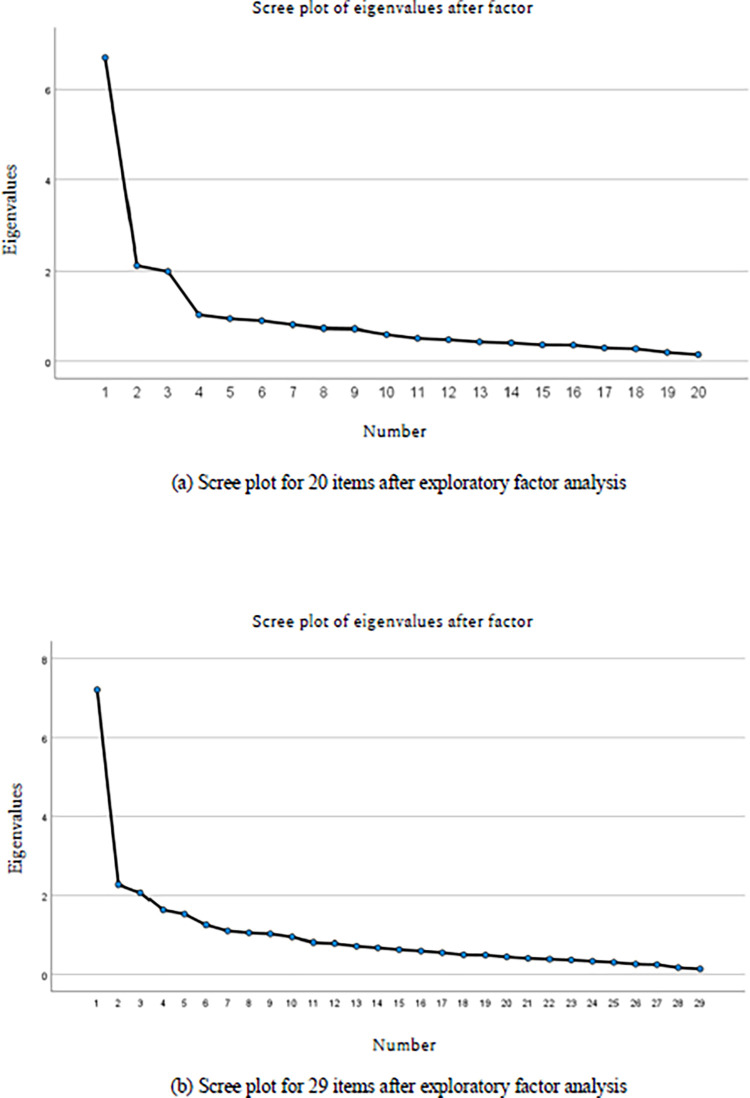
Comparison of scree plot after exploratory factor analysis.

Another round of exploratory factor analysis using principal factor and Promax rotation with 20 items and 29 items (S4 Table) were performed. There was significant positive correlation between the first factor and the second factor for both 20 items (r = 0.56) and 29 items (r = 0.58). When we compare the scree plot, the “break” in the scree plot for 20 items after exploratory factor analysis showed steeper bend between the third factor and the fourth factor (Fig 1), indicating that a three-factor structure would be an appropriate and data-driven solution. The scree plot for the 29 items after exploratory factor analysis yielded one dominant domain, but did not show a clear three-factor solution (Fig 1).

The items and data were carefully analyzed, and the decision was made to eliminate 11 items with cut-off of less than 0.3 using a data-driven approach. The items excluded were “2. introduce themselves”, “7. record confidentiality”, “9. consent to procedures”, “21. verbal abuse”, “22. physical abuse”, “23. bribes”, “26. trust”, “28. clean”, “29. water”, “30. electricity”, and “31. safe”. The decision was made based on the following reasons: (1) all items had low factor loadings of less than 0.3; (2) item #7, 21, 22, 23, 29, 30, and 31 had particularly high mean (+1SD) greater than 2.9 in an upper limit of 3; (3) item #2 had low I-CVI (0.75); (4) items #28, 29, 30, and 31 are theoretically classified into health facility environmental dimension of quality of care, which may be conceptualized differently from experience of care (Table 3).

**Table 3:** Items for person-centered maternity care scale.

Domain	Question	Referred to in text as	Disposition	Reasons
Dignity and respect	#4: Did the doctors, nurses, or other staff at the facility treat you with respect?	Treated with respect	Retained	
	#5: Did the doctors, nurses, and other staff at the facility treat you in a friendly manner?	Friendly	Retained	
	#6: During examinations in the labor room, were you covered up with a cloth or blanket or screened with a curtain so that you did not feel exposed?	Visual privacy	Retained	
	#7: Do you feel like your health information was or will be kept confidential at this facility?	Record confidentiality	Deleted	Low factor loading of less than 0.3 High mean (+1SD) of greater than 2.9
	#21: Did you feel the doctors, nurses, or other health providers shouted at you, scolded, insulted, threatened, or talked to you rudely?	Verbal abuse	Deleted	Low factor loading of less than 0.3 High mean (+1SD) of greater than 2.9
	#22: Did you feel like you were treated roughly like pushed, beaten, slapped, pinched, physically restrained, or gagged?	Physical abuse	Deleted	Low factor loading of less than 0.3 High mean (+1SD) of greater than 2.9
Communication and autonomy	#2: During your time in the health facility did the doctors, nurses, or other health care providers introduce themselves to you when they first came to see you?	Introduce self	Deleted	Low factor loading of less than 0.3 Low I-CVI
	#3: Did the doctors, nurses, or other health care providers call you by your name?	Called by name	Retained	
	#8: Did you feel like the doctors, nurses or other staff at the facility involved you in decisions about your care?	Involvement in care	Retained	
	#9: Did the doctors, nurses or other staff at the facility ask your permission/consent before doing procedures on you?	Consent to procedures	Deleted	Low factor loading of less than 0.3
	#10: During the delivery, do you feel like you were able to be in the position of your choice?	Delivery position choice	Retained	
	#11: Did the doctors, nurses or other staff at the facility speak to you in a language you could understand?	Language	Retained	
	#12: Did the doctors and nurses explain to you why they were doing examinations or procedures on you?	Explain exams/procedures	Retained	
	#13: Did the doctors and nurses explain to you why they were giving you any medicine?	Explain medicines	Retained	
	#16: Did you feel you could ask the doctors, nurses or other staff at the facility any questions you had?	Able to ask questions	Retained	
Supportive care	#1: How did you feel about the amount of time you waited? Would you say it was very short, somewhat short, somewhat long, or very long?	Time to care	Retained	
	#14: Did the doctors and nurses at the facility talk to you about how you were feeling?	Talk about feeling	Retained	
	#15: Did the doctors, nurses or other staff at the facility try to understand your anxieties and fears?	Support anxiety	Retained	
	#17: Were you allowed to have someone you wanted to stay with you during labor?	Labor support	Retained	
	#18: Were you allowed to have someone you wanted to stay with you during delivery?	Delivery support	Retained	
	#19: When you needed help, did you feel the doctors, nurses or other staff at the facility paid attention?	Attention when need help	Retained	
	#20: Do you feel the doctors or nurses did everything they could to help control your pain?	Control pain	Retained	
	#23: Did the doctors, nurses or other staff at the facility ask you or your family for money other than the official cost	Bribes	Deleted	Low factor loading of less than 0.3 High mean (+1SD) of greater than 2.9
	#24: Do you think there was enough health staff in the facility to care for you?	Enough staff	Retained	
	#25: Did you feel the doctors, nurses or other staff at the facility took the best care of you?	Took best care	Retained	
	#26: Did you feel you could completely trust the doctors, nurses or other staff at the facility with regards to your care?	Trust	Deleted	Low factor loading of less than 0.3
	#27: Thinking about the labor and postnatal wards, did you feel the health facility was crowded?	Crowded	Retained	
	#28: Thinking about the wards, washrooms and the general environment of the health facility, will you say the facility was very clean, clean, dirty, or very dirty?	Clean	Deleted	Low factor loading of less than 0.3 Theoretically different dimension
	#29: Was there water in the facility?	Electricity	Deleted	Low factor loading of less than 0.3 High mean (+1SD) of greater than 2.9 Theoretically different dimension
	#30: Was there electricity in the facility?	Water	Deleted	Low factor loading of less than 0.3 High mean (+1SD) of greater than 2.9 Theoretically different dimension
	#31: In general, did you feel safe in the health facility?	Safe	Deleted	Low factor loading of less than 0.3 High mean (+1SD) of greater than 2.9 Theoretically different dimension

Exploratory factor analysis of 20 items yielded three factors, 12 items loaded on the first factor, six items on the second factor, and two items on the third factor (Table 4). The three factors were named PCMC factor 1, PCMC factor 2, and PCMC factor 3 namely birth companionship, respectively. The items loading on each of the three factors did not represent clear conceptual domains, because the factors extracted included a mix of items from each of the original domains. For example, the first factor included “11. language”, “13. call by name”, “16. able to ask questions”, which conceptually should have loaded on the second factor, and “14. talk about feeling”, “19. attention when needed help”, “20. control pain”, which conceptually should have loaded on the third factor.

**Table 4:** Exploratory factor analysis result of 20 items.

The data driven sub-scales	Items	Factor 1	Factor 2	Factor 3	Theoretical factor structure
PCMC factor 1	16 Able to ask questions	**.92**	-.15	-.07	Communication and autonomy
	5 friendly manners	**.89**	-.04	-.10	Dignity and respect
	11 Language	**.67**	.09	.01	Communication and autonomy
	14 Talk about feeling	**.64**	.07	-.11	Supportive care
	19 Attention when needed help	**.64**	.13	.16	Supportive care
	4 Respect	**.64**	.22	-.06	Dignity and respect
	10 Delivery position choice	**.62**	-.24	.28	Communication and autonomy
	27 crowded	**.55**	-.39	-.06	Supportive care
	20 Control pain	**.47**	.26	-.05	Supportive care
	15 Support anxiety	**.46**	.19	.03	Supportive care
	3 Call by name	**.45**	.25	.00	Communication and autonomy
	1 Time to care	**.38**	-.26	.04	Supportive care
PCMC factor 2	8 Involvement in care	-.22	**.80**	-.04	Communication and autonomy
	24 Enough staff	-.08	**.66**	.02	Supportive care
	13 Explain medicine	.20	**.62**	.04	Communication and autonomy
	12 Explain procedures	.18	**.57**	.11	Communication and autonomy
	25 Took best care	.16	**.52**	-.02	Supportive care
	6 Privacy	-.18	**.34**	-.08	Dignity and respect
PCMC factor 3 (Birth companionship)	18 Delivery companion	-.01	.02	**.93**	Supportive care
	17 Labor companion	-.05	-.02	**.90**	Supportive care
	Correlation between factors	Ⅰ	Ⅱ	Ⅲ	
	Ⅰ	ー	.56*	.06	
	Ⅱ		ー	.03	
	Ⅲ			ー	

Principal factor, Promax rotation.

* *P* < 0.01.

We, therefore, regrouped the retained items into three conceptual domains drawn from the “experience of care” dimension of the WHO Quality of Care Framework. However, some items loaded negatively on the theoretically derived domain and positively on the data-driven domain. The original three-factor structure was not reproduced from Cambodian data. Instead, the distribution of the items, cultural rationale, and the judgment from the tool developer was considered.

(3) Internal consistency

The 20-item Kh-PCMC scale had a Cronbach’s alpha of 0.86, suggesting good internal consistency. Cronbach’s alphas of the three subscales: PCMC factor 1, PCMC factor 2, and PCMC factor 3 were 0.85, 0.76, and 0.91, respectively (Table 5). In contrast the Cronbach’s alpha for the theoretically derived 30-item scale is 0.85, with Cronbach’s alphas 0.47, 0.77, and 0.68 for the dignity and respect, communication and autonomy, and supportive care subscales respectively.

**Table 5:** Lists of reliability and validity.

		Full PCMC scale (20 items)	Subscale		
			Dignity and respect (12 items)	Communication and autonomy (6items)	Supportive care (2 items)
Internal consistency (N = 300)		α = 0.86	α = 0.85	α = 0.76	α = 0.91
Criterion validity (N = 300)					
	Satisfaction with care	0.25***	0.60***	0.47***	-0.13*
	Quality of care rating	0.59***	0.31***	0.15**	-0.11
	Future intention to give birth in the same facility	-0.07	-0.28	-0.08	-0.04

*** *P* < 0.001 (2-tailed) ** *P* < 0.01 (2-tailed) * *P* < 0.05 (2-tailed).

(4) Criterion validity

The 20-item Kh-PCMC full scale score was significantly correlated with satisfaction with care (r = 0.249, P < 0.001) and quality of care rating (r = 0.593, P < 0.001) (Table 5).

(5) Cross-cultural validity

The preliminary work for cultural translation and adaptation supported acceptable cross-cultural validity. The correlation between the 20-item and 30-item scale is 0.99 (*P* < 0.001).

### Score distribution of 20-item Kh-PCMC scale

The 20-item Kh-PCMC scale with a four-point frequency response ranging from 0 to 3 (“0 = No, never”, “1 = Yes, a few times”, “2 = Yes, most of the time”, “3 = Yes, all the time”) was proposed. The item ratings were aggregated to scale scores by summing each item. The total possible summative score ranged from 0 to 60, with scores representing better person-centered maternity care. The mean scores of 20 item Kh-PCMC scale for this sample is 44.25 (SD = 8.68). The mean scores of the three subscales are 25.68 (SD = 5.87), 13.40 (SD = 3.79), and 5.18 (SD = 1.72), respectively. Standardized scores ranging from 0 to 100 are 73.75 for full the score, and 71.33, 74.44, and 86.33 for the three subscales, respectively (Table 6).

**Table 6:** Distribution of 20 item Kh-PCMC scale and subscales in Cambodia (n = 300).

	Number of items	Mean raw scores	SD	Min	Max	Possible range of summative scores	Standardized scores	Possible range of standardized scores
20-item PCMC Scale	20	44.25	8.68	26	60	0 to 60	73.75	0 to 100
PCMC sub scale 1	12	25.68	5.87	13	36	0 to 36	71.33	0 to 100
PCMC sub scale 2	6	13.40	3.79	4	18	0 to 18	74.44	0 to 100
PCMC sub scale 3	2	5.18	1.72	0	6	0 to 6	86.33	0 to 100

## Discussion

The present study provided evidence that the 20-item Kh-PCMC scale is a valid and reliable tool to measure women’s experience of maternity care among Cambodian postpartum women in facility settings. The preliminary work towards the development of this scale including cognitive interviewing and expert review ensured good content validity and acceptable cross-cultural validity [25]. The S-CVI/Avg of 0.96 also showed high content validity. The 20-item Kh-PCMC scale has high internal consistency reliability with a Cronbach’s alpha of 0.86 for the full scale and 0.76–0.91 for the subscales. Similar results were found with the Kenyan version, namely, high internal consistency reliability with a Cronbach’s alpha of 0.88 for a rural sample, 0.83 for an urban sample, and 0.86 for a combined sample [16], and in Indian version which had a Cronbach’s alpha of 0.85 [17]. In turn, hypothesis testing found correlations between the 20-item Kh-PCMC scale and reference measures, indicating acceptable criterion validity within this field where gold standards are not available. This is consistent with the Kenyan version which showed that a higher PCMC score was associated with increasing satisfaction with care and rating of quality of care [16].

### Factor interpretation

Due to the potential cultural and social differences, it is necessary to validate the PCMC scale in a different context. Nineteen of 30 items were common across Kenya (Africa), India (South Asia), and Cambodia (Southeast Asia), which enable meaningful international comparisons among very different settings. Our exploration found the items loading on each subscale differed from the original version, while the overall PCMC concept remained similar. For example, the first factor (dignity and respect) included “3. call by name”, “10. delivery position choice”, “11. language”, and “16. able to ask questions”, which conceptually should have loaded on the second factor (communication and autonomy), and “14. talk about feelings”, “19. attention when needed help”, and “20. control pain”, which conceptually should have loaded on the third factor (supportive care). Our finding may indicate that differing local contexts and cultures influenced the women’s experience of received care, which influenced item loading.

There are four potential explanations for the difference in item location. First, it is probably attributable to the overarching themes of the PCMC that produce meaningful interactions between the subscales. Our finding showed a significant positive correlation between the subscales of “dignity and respect” and “communication and autonomy” (r = 0.51). This is consistent with the original version in which the subscales were shown to be strongly correlated with each other, with correlation coefficients (r) ranging from 0.53 to 0.63, and with the main scale (r = 0.75, 0.86, and 0.9 for dignity and respect, communication and autonomy, and supportive care, respectively) [16]. The original PCMC scale was developed as a theory-based practical tool that can be easily administered in various contexts [16]. A recent study proposed a unidimensional 13-items PCMC short scale using a data-driven approach that could be applied to multiple settings [40]. Thus, there may be flexibility of which items fits which subscales according to the context.

Second, the difference in item location could be explained by contextual difference. This is supported by the previous validation studies which showed that the factor loading was different between urban and rural populations within Kenya [16], and the factor loading from Indian data was also different from the conceptual domains [17]. Because the total number of respondents were 1,407 in Kenya [16], and 2,018 in India [17], the difference was not due to sampling issues. Rather, the factor structure may differ across different contexts and different sub-populations. In that sense, our findings reflected local reality where the concept of person-centered maternity care was not yet familiar and not commonly practiced [41]. The original PCMC scale consists of three conceptual domains, however, there may not have been clear differences among “dignity and respect,” “community and autonomy,” and “supportive care” for our respondents under the current situation in Cambodia.

Third, language issue related to the equivalence of translation may be another potential reason. The PCMC scale was validated in Kenya and India, where English is one of the official languages and the interviews were conducted in English, Swahili, and Luo in Kenya, in Hindi in India, respectively. On the other hand, in Cambodia, the official language and interview language were in Khmer. The limited vocabulary of the Khmer language and the issue that English is not commonly used in the country may have influenced the limited nuanced translation from English into Khmer. This is consistent with a recent study from Cambodia in which the translation from English to Khmer was a big challenge due to unfamiliarity with nuanced technical jargon in the cultural and linguistic settings [42]. This is also consistent with other studies that have shown how terms can be influenced by culture and render translations conceptually different [21,43]. The language barrier is one of the limitations of any cross-cultural study.

Fourth, comprehension errors among Cambodian postpartum women may have affected the quality of data. Thus, the obtained data from the respondents may have influenced the results of factor analysis.

Another potential issue is whether the third factor holds as a factor, because there were only two items of labor companion and delivery companion in that factor. This can be justified by cultural importance. In Cambodia, the cultural values based on the mixture of Animism, Hinduism, and Buddhism are strongly reflected in the perspectives and behaviors of women during maternity such as “reincarnation” and “karma” [44]. The items included in the first factor (PCMC subscale 1) may reflect items in which women felt the medical staff did something good to/for them. In the Cambodian context, this was probably attributed to karma, as it is also considered as good karma to let the others do good deeds. This is empirically supported by a JICA project (2010–2015) in which when introducing the new concept of midwifery care, Cambodian medical staff incorporated the concept in connection with the heart of mercy [41]. Two items of labor companion and delivery companion that loaded on the third factor (PCMC subscale 3) were both related to family presence. Our result agrees with a previous report which found that family-like care was a reasonable way for Cambodian medical staff to understand the concept of person-centered maternity care [45]. Cambodian people attach great importance to the family which is reflective of its collectivistic culture [46]. This is consistent with the evidence from Nigeria and Uganda where women “desired midwives who acted as “mums” to them, who warmly received them, and who provided reassurance and encouragement to give birth well” [47]. Further, our result is consistent with a unique Cambodian contextual feature that nursing tasks are shared among doctors, nurses, and patient families. Many non-invasive nursing cares including bedside hygiene, bathing, and changing sanitary napkins, were normally provided by the patient family [48]. Cambodian women are more likely to seek emotional support and reassurance from their family. Therefore, even though it only consisted of two items, the third factor was retained as a factor that reflects a context where family support is important.

### Item interpretation

The four items related to the health facility environment (clean, water, electricity, and safe) did not load well and were eliminated from the 20-item Kh-PCMC scale, which was included in the “supportive care” sub-scale in the Kenyan validation. This is consistent with previous PCMC validation studies in which three items related to the health facility environment (water, electricity, and crowding) were removed from the version in India [17] and also from the 13-item short scale due to poor factor loading [40]. In the original PCMC scale, items related to the health facility environment were retained because they are conceptually and empirically important aspects of person-centered care [49,50], and because the independent health facility environment subscale had low reliability [16]. On the contrary, the health facility environment is theoretically an independent dimension from “experience of care” within the WHO Quality of Care Framework [1]. Thus, the poor loading of items related to the health facility environment may be attributable to this theoretical difference. The facility environment is a foundational requirement in care settings that influences experience of care but may be distinct from experience of care.

Two items related to disrespect and abuse (verbal abuse and physical abuse) did not load well and were eliminated from the 20-item Kh-PCMC scale, which were included in “dignity and respect” sub-scale in the Kenyan validation. In Kenya, the item physical abuse had poor loading but was retained due to conceptual and empirical significance [16]. The poor loadings of items related to abuse were likely due to the low prevalence of verbal and physical abuse in Cambodia. Comparing to other available studies, the percentage of verbal abuse was 4% and that of physical abuse was 3% in Cambodia, 10% and 4% in rural Kenya, 18% and 1% in urban Kenya, 13% and 4% in Ghana, and 19% and 3% in India, respectively [19]. Worldwide, there have been rising reports of disrespect and abuse in maternity care in institutional settings [51–53], hence eliminating disrespect and abuse during the childbirth is an urgent problem [54]. Previous research has shown that self-reported measures were likely to underreport instances of disrespect and abuse during childbirth compared to direct observation, because it becomes internalized and normalized for both care provider and care receiver [55]. Thus, direct observation may be a more effective way to investigate the reality of disrespect and abuse.

Item related to consent to procedure did not load well and was eliminated from the 20-item Kh-PCMC scale, which was included in “communication and autonomy” sub-scale in the Kenyan validation. The results of the preliminary study for cultural translation and adaptation of PCMC scale in Cambodia suggested that some global concepts of PCMC may not resonate with local women’s views and perceptions on PCMC [25]. For example, some Cambodian women did not distinguish well between the concepts of consent to procedures and explanation of procedures. This is particularly so, where women are in a society noted for blind adherence to expert suggestion, implicit consent, poor awareness of alternative options, and gender norms and social hierarchy between medical staff and care receivers. And due to Cambodian women’s way of cognition, we often needed to add explanation to the structured questions. This may have introduced some variation in how women understood the questions if the explanations were not the same for everyone, hence, it may have influenced the quality of data.

### Limitations and strengths

There are several limitations in the present study. First, the sample was not generalizable to all Cambodia. Future studies should aim to include samples from all regions and all levels of health facilities and private clinics.

Second, limited resources may have influenced quality of data. Although our translators, expert panels, and data collectors were not experienced professionals, we did our utmost to proceed with the study within the limited available resources and under COVID-19 restrictions.

Third, social desirability bias is a concern as the interviews were conducted in the maternity ward before discharge. Previous studies suggested that women were less likely to report negative experiences inside health facilities [56,57]. In addition, other research suggested that women were more likely to report their experiences positively when interviewed earlier in the postpartum period due to the joy of having just delivered a baby [58,59]. The mean postpartum length of our respondents was 2.5 days, which is similar to within 48 hours in the study from India, but shorter than the within nine weeks period used in the study from rural Kenya, within one week in urban Kenya, and within eight weeks in the Ghanaian study [19]. On the contrary, another study suggests that two to seven days of health facility stay were associated with a significantly decreased PCMC score, due to the increasing probability of experiencing poor person-centered care during the stay [60]. In this sense, the PCMC score found in this study is likely to overestimate actual levels. Relatedly, as noted in another paper [25], it was difficult to conduct an interview with a woman in private. This is because it was not culturally appropriate to keep her family member(s) away for an early postpartum woman. And it is also because a woman took it for granted that family member(s) would be present. Women may be inclined to provide socially desirable responses when family members were present.

Data collection was conducted by having an interviewer read out each question in person. This data collection mode was not only time-consuming and expensive, but also lead to biased or induced response. Nevertheless, the data collection mode is probably the most practical way to obtain accurate data from Cambodian women. This is because all 20 participants in Phase 1 expressed their preference to have an interviewer read the questions and response to them. When asking socio-culturally sensitive questions in a survey targeting women (e.g. person-centered maternity care), it should be necessary to pay the utmost consideration to the way of asking question. That will help a survey avoid ensure women’ comfort during an interview particularly during immediate postpartum period. It is also important for researchers to interpret the data assuming possible biases and limitations in women’ responses, when conducting interviews not at their home but at health facilities.

Fourth, similar to previous studies [22,61], balancing content validity and maintaining linguistic and statistical accuracy was a big challenge. The content validity is the single most important psychometric property of a questionnaire [28]. Only when there is good content validity, can the questionnaire can be considered successful, and the rest of the psychometric properties become useful [36]. In the present study, the content validity of the 31-item Kh-PCMC scale was assured by literature review, expert review, cognitive interviewing, and CVI. However, 11 items were excluded due to poor psychometric properties. We were therefore concerned about eliminating items that matter to Cambodian women during childbirth. The present study used a cut-off of 0.3 [34] for a more data-driven approach, while the validation in Kenya and India used conservative and inclusive decisions, and a relaxed cut-off of 0.1 to retain some key items such as verbal and physical abuse [16,17]. Given the potential importance of some excluded items and the limited generalizability of the validation sample, we recommend future studies include all 31 PCMC items to reassess the psychometric properties in more diverse samples. In addition, without qualitative research in Cambodia for an item generation stage, it is possible that we excluded some aspects of what matters to Cambodian women during childbirth. Future qualitative research is required to comprehensively capture PCMC for the Cambodian population.

Despite these limitations, there are strengths of the present study. First of all, to the best of our knowledge, this is the first reliable and valid instrument to quantitatively measure women’s experience of care received during childbirth in Cambodia. Since we have an assumption that potential cultural and social differences may influence the conceptualization of person-centered maternity care, the notable strength of the present study is to emphasize cultural context, language, and local practices for use in Cambodia.

Second, a recent qualitative evidence synthesis suggested that what matters to women during childbirth were consistent across many settings, albeit that the evidence to support this claim thus far has come from only one continent of the world (Africa) [62]. The previous validation studies of the PCMC scale also called for further validation in additional settings including Southeast Asian populations with a data-driven approach [40]. Therefore, we believe this is the first significant response from Cambodia using such a data-driven approach.

Third, developing the global standard to measure person-centered maternity care is an urgent priority in this area [16] and, the present study contributed to additional validation of the PCMC scale in the Asian context to facilitate meaningful international comparison.

## Conclusion

In summary, in the present study, the 20-item Kh-PCMC scale was developed and validated. The translation and pretesting process was optimized to achieve acceptable conceptual and semantic equivalence between the original PCMC scale and the 20-item Kh-PCMC scale. The findings from the psychometric analysis supported acceptable content validity, construct validity, criterion validity, and high internal consistency reliability of the 20-item Kh-PCMC scale.

The present study provides an effective tool to quantitatively measure women’s childbirth experiences to better understand the quality of intrapartum care, and to identify women’s intrapartum needs from their perspective for quality improvement in Cambodia. In addition, the 20-item Kh-PCMC scale will facilitate further research in Cambodia to allow comparisons across settings and time, statistical analysis to examine the determinants and health outcomes of care during childbirth, and routine monitoring and evaluation of interventions and projects based on the WHO recommendation on intrapartum care for a positive childbirth experience [2].

However, because culture is not static or identical across regions, but changes over time and place, further studies are needed to refine the PCMC scale and perhaps formulate additional items specific to the Cambodian cultural context. Continuous effort should be taken to finetune the instrument over time to meet the changing needs of Cambodian women.

## Supporting information

S1 Table Mean (SD) of 31 item Kh-PCMC scale. (DOCX)

S2 Table Distribution of full PCMC scale and subscales in Cambodia (n = 300). (DOCX)

S3 Table Distribution of PCMC variables. (DOCX)

S4 Table Exploratory factor analysis result of 29 items of the Kh-PCMC scale. (DOCX)

S5 Table Kh-PCMC scale. (DOCX)
