# Correction: Validation of the person-centered maternity care scale at governmental health facilities in Cambodia

**DOI:** 10.1371/journal.pone.0335546

**Published:** 2025-10-27

**Authors:** Yuko Takahashi Naito, Rieko Fukuzawa, Togoobaatar Ganchimeg, Asako Takekuma Katsumata, Patience A. Afulani, Rattana Kim, Hirotsugu Aiga

The authors are listed out of order. Please view the correct author order, affiliations, and citation here:

Yuko Takahashi Naito^1^, Rieko Fukuzawa^2^, Togoobaatar Ganchimeg^2^, Asako Takekuma Katsumata^2^, Patience A. Afulani^3^, Rattana Kim^5^, Hirotsugu Aiga^4^

**1** Graduate School of Comprehensive Human Sciences, University of Tsukuba, Tsukuba, Japan, **2** Faculty of Medicine, University of Tsukuba, Ibaraki, Japan, **3** Departments of Epidemiology & Biostatistics and Obstetrics, Gynecology, & Reproductive Sciences, University of California, San Francisco (UCSF), San Francisco, CA, United States of America, **4** School of Tropical Medicine and Global Health, Nagasaki University, Nagasaki, Japan, **5** National Maternal and Child Health Center, Phnom Penh, Cambodia
