# Supplementary material for: Validation of the person-centered maternity care scale at governmental health facilities in Cambodia
Source: PLoS One. 2023 Jul 6;18(7):e0288051. doi: 10.1371/journal.pone.0288051 (PMC10325110; doi:10.1371/journal.pone.0288051)
Supplement: S1 Table — (DOCX) [file pone.0288051.s001.docx]

**S1 Table.** Mean (SD) of 31 item Kh-PCMC scale

| # | Item | Mean | SD |
| --- | --- | --- | --- |
| 1 | Time to care | 2.26 | 0.04 |
| 2 | Introduce self | 0.26 | 0.04 |
| 3 | Called by name | 1.62 | 0.06 |
| 4 | Treated with respect | 2.28 | 0.03 |
| 5 | Friendly | 2.10 | 0.04 |
| 6 | Visual privacy | 2.86 | 0.02 |
| 7 | Record confidentiality | 2.90 | 0.02 |
| 8 | Involvement in care | 2.16 | 0.07 |
| 9 | Consent to procedures | 2.72 | 0.03 |
| 10 | Delivery position choice | 2.26 | 0.05 |
| 11 | Language | 2.26 | 0.04 |
| 12 | Explain exams/procedures | 2.06 | 0.06 |
| 13 | Explain medicines | 1.63 | 0.08 |
| 14 | Talk about feeling | 2.04 | 0.04 |
| 15 | Support anxiety | 2.31 | 0.04 |
| 16 | Able to ask questions | 2.09 | 0.04 |
| 17 | Labor support | 2.62 | 0.05 |
| 18 | Delivery support | 2.56 | 0.05 |
| 19 | Attention when need help | 2.17 | 0.04 |
| 20 | Control pain | 2.09 | 0.06 |
| 21 | Verbal abuse | 2.93 | 0.02 |
| 22 | Physical abuse | 2.93 | 0.02 |
| 23 | Bribes | 2.96 | 0.01 |
| 24 | Enough staff | 2.33 | 0.05 |
| 25 | Took best care | 2.36 | 0.04 |
| 26 | Trust | 2.59 | 0.03 |
| 27 | Crowded | 2.18 | 0.05 |
| 28 | Clean | 1.96 | 0.02 |
| 29 | Electricity | 2.91 | 0.02 |
| 30 | Water | 2.96 | 0.01 |
| 31 | Safe | 2.90 | 0.02 |
