# Supplementary material for: Validation of the person-centered maternity care scale at governmental health facilities in Cambodia
Source: PLoS One. 2023 Jul 6;18(7):e0288051. doi: 10.1371/journal.pone.0288051 (PMC10325110; doi:10.1371/journal.pone.0288051)
Supplement: S2 Table — (DOCX) [file pone.0288051.s002.docx]

**S2 Table.** Distribution of full PCMC scale and subscales in Cambodia (n=300)

|  | Number of items | Mean raw scores | SD | Min | Max | Possible range of summative scores | Standardized scores | Possible range of standardized scores |
| --- | --- | --- | --- | --- | --- | --- | --- | --- |
| Full PCMC Scale | 30 | 69.32 | 9.47 | 48 | 89 | 0 to 90 | 77.02 | 0 to 100 |
| Dignity and respect | 6 | 16.01 | 1.53 | 8 | 18 | 0 to 18 | 88.94 | 0 to 100 |
| Communication and autonomy | 9 | 15.43 | 3.92 | 6 | 24 | 0 to 27 | 57.15 | 0 to 100 |
| Supportive Care | 15 | 36.26 | 4.38 | 24 | 44 | 0 to 45 | 80.58 | 0 to 100 |
