# Supplementary material for: Validation of the person-centered maternity care scale at governmental health facilities in Cambodia
Source: PLoS One. 2023 Jul 6;18(7):e0288051. doi: 10.1371/journal.pone.0288051 (PMC10325110; doi:10.1371/journal.pone.0288051)
Supplement: S3 Table — (DOCX) [file pone.0288051.s003.docx]

**S3 Table.** Distribution of PCMC variables

| PCMC variable | | Number | Percent |
| --- | --- | --- | --- |
| #1. Did you feel to wait long or short from when you arrived to when you received care? | |  |  |
|  | 0. very short | 118 | 39.20 |
|  | 1. somewhat short | 142 | 47.20 |
|  | 2. somewhat long | 39 | 13.00 |
|  | 3. very long | 1 | 0.30 |
|  |  |  |  |
| #2. During your time in the health facility did the medical staff introduce themselves to you when they first came to see you? For example, their name or profession. | |  |  |
|  | 0 No, none of them | 259 | 86.00 |
|  | 1 Yes, a few of them | 19 | 6.30 |
|  | 2 Yes, most of them | 6 | 2.00 |
|  | 3 Yes, all of them | 16 | 5.30 |
|  |  |  |  |
| #3. Did the medical staff call you by your name? | |  |  |
|  | 0 No, never | 49 | 16.30 |
|  | 1 Yes, a few times | 90 | 29.90 |
|  | 2 Yes, most of the time | 88 | 29.20 |
|  | 3 Yes, all the time | 73 | 24.30 |
|  |  |  |  |
| #4. Did the medical staff at the facility treat you with respect? | |  |  |
|  | 0 No, never | 1 | 0.30 |
|  | 1 Yes, a few times | 16 | 5.30 |
|  | 2 Yes, most of the time | 180 | 59.80 |
|  | 3 Yes, all the time | 103 | 34.20 |
|  |  |  |  |
| #5. Did the medical staff at the facility treat you in a friendly manner? | |  |  |
|  | 0 No, never | 1 | 0.30 |
|  | 1 Yes, a few times | 56 | 18.60 |
|  | 2 Yes, most of the time | 154 | 51.20 |
|  | 3 Yes, all the time | 89 | 29.60 |

| PCMC variable | | Number | Percent |
| --- | --- | --- | --- |
| #6. During examinations in the labor room (for example, pelvic examination), were you covered up with a cloth or blanket or screened with a curtain? | |  |  |
|  | 0 No, never | 1 | 0.30 |
|  | 1 Yes, a few times | 2 | 0.70 |
|  | 2 Yes, most of the time | 36 | 12.00 |
|  | 3 Yes, all the time | 261 | 86.70 |
|  |  |  |  |
| #7. Do you feel like your health information was kept confidential at this facility? For example, the information on the medical record. | |  |  |
|  | 0 No, never | 2 | 0.70 |
|  | 1 Yes, a few times | 2 | 0.70 |
|  | 2 Yes, most of the time | 22 | 7.30 |
|  | 3 Yes, all the time | 250 | 83.10 |
|  | 4 Did not know it was kept confidential | 24 | 8.00 |
|  |  |  |  |
| #8. Did you feel like the medical staff at the facility considered your ideas in decisions about your care? For example, can you decide for yourself whether you want to have a natural or caesarean section? | |  |  |
|  | 0 No, never | 56 | 18.60 |
|  | 1 Yes, a few times | 11 | 3.70 |
|  | 2 Yes, most of the time | 62 | 20.60 |
|  | 3 Yes, all the time | 157 | 52.20 |
|  | 4 Did not have to make any decisions | 0 | 0.00 |
|  | 5 preferable to follow doctors | 14 | 4.70 |
|  |  |  |  |
| #9. Did the medical staff at the facility ask your permission/consent before doing procedures on you? For example, pelvic examination and episiotomy? | |  |  |
|  | 0 No, never | 1 | 0.30 |
|  | 1 Yes, a few times | 9 | 3.00 |
|  | 2 Yes, most of the time | 63 | 20.90 |
|  | 3 Yes, all the time | 227 | 75.40 |

| PCMC variable | | Number | Percent |
| --- | --- | --- | --- |
| #10. During the delivery, do you feel like you were able to be in your favorite free position? | |  |  |
|  | 0 No, never | 17 | 5.60 |
|  | 1 Yes, for a short time | 36 | 12.00 |
|  | 2 Yes, most of the time | 100 | 33.20 |
|  | 3 Yes, all the time | 143 | 47.50 |
|  | 4 No choice other than following doctors​ | 4 | 1.30 |
|  |  |  |  |
| #11. Did the medical staffs at the facility speak to you in a language you could understand? | |  |  |
|  | 0 No, never | 1 | 0.30 |
|  | 1 Yes, a few times | 23 | 7.60 |
|  | 2 Yes, most of the time | 173 | 57.50 |
|  | 3 Yes, all the time | 103 | 34.20 |
|  |  |  |  |
| #12. Did the medical staff explain to you the objectives or reasons why they were doing examinations or procedures on you? For example, pelvic examination or fetal heart rate monitoring. | |  |  |
|  | 0 No, never | 38 | 12.60 |
|  | 1 Yes, a few times | 21 | 7.00 |
|  | 2 Yes, most of the time | 127 | 42.20 |
|  | 3 Yes, all the time | 114 | 37.90 |
|  |  |  |  |
| #13. Did the medical staff explain to you why they were giving you any medicine? | |  |  |
|  | 0 No, never | 95 | 31.60 |
|  | 1 Yes, a few times | 40 | 13.30 |
|  | 2 Yes, most of the time | 46 | 15.30 |
|  | 3 Yes, all the time | 118 | 39.20 |
|  | 4 Did not get any medicine | 1 | 0.30 |

| PCMC variable | | Number | Percent |
| --- | --- | --- | --- |
| #14. Did the medical staff at the facility talk to you about how you were feeling? | |  |  |
|  | 0 No, never | 28 | 9.30 |
|  | 1 Yes, a few times | 7 | 2.30 |
|  | 2 Yes, most of the time | 35 | 11.60 |
|  | 3 Yes, all the time | 230 | 76.40 |
|  |  |  |  |
| #15. Did the medical staff at the facility try to understand your anxieties and fears? | |  |  |
|  | 0 No, never | 2 | 0.70 |
|  | 1 Yes, a few times | 17 | 5.60 |
|  | 2 Yes, most of the time | 166 | 55.10 |
|  | 3 Yes, all the time | 114 | 37.90 |
|  | 4 I did not have any anxieties or fears | 1 | 0.30 |
|  |  |  |  |
| #16. Did you feel you could ask t the medical staffs at the facility any questions you had? | |  |  |
|  | 0 No, never | 2 | 0.70 |
|  | 1 Yes, a few times | 57 | 18.90 |
|  | 2 Yes, most of the time | 152 | 50.50 |
|  | 3 Yes, all the time | 89 | 29.60 |
|  |  |  |  |
| #17. Were you allowed to have someone you wanted to stay with you during labor? | |  |  |
|  | 0 No, never | 24 | 8.00 |
|  | 1 Yes, a few times | 5 | 1.70 |
|  | 2 Yes, most of the time | 32 | 10.60 |
|  | 3 Yes, all the time | 238 | 79.10 |
|  | 4 I did not want someone to stay with me | 1 | 0.30 |
|  |  |  |  |
| #18. Were you allowed to have someone you wanted to stay with you during delivery? | |  |  |
|  | 0 No, never | 28 | 9.30 |
|  | 1 Yes, a few times | 7 | 2.30 |
|  | 2 Yes, most of the time | 35 | 11.60 |
|  | 3 Yes, all the time | 230 | 76.40 |
|  | 4 I did not want someone to stay with me | 0 | 0 |

| PCMC variable | | Number | Percent |
| --- | --- | --- | --- |
| #19. When you needed help, did you feel the medical staff at the facility respond to needs? | |  |  |
|  | 0 No, never | 7 | 2.30 |
|  | 1 Yes, a few times | 22 | 7.30 |
|  | 2 Yes, most of the time | 183 | 60.80 |
|  | 3 Yes, all the time | 88 | 29.20 |
|  |  |  |  |
| #20. Do you feel the medical staff did everything they could to help control your pain? | |  |  |
|  | 0 No, never | 26 | 8.60 |
|  | 1 Yes, a few times | 45 | 15.00 |
|  | 2 Yes, most of the time | 118 | 39.20 |
|  | 3 Yes, all the time | 97 | 32.20 |
|  | 4 No pain | 14 | 4.70 |
|  |  |  |  |
| #21. Did you feel the medical staff shouted at you, scolded, insulted, threatened, or talked to you rudely? | |  |  |
|  | 0 No, never | 289 | 96.00 |
|  | 1 Yes, once | 5 | 1.70 |
|  | 2 Yes, a few times | 3 | 1.00 |
|  | 3 Yes, many time | 3 | 1.00 |
|  |  |  |  |
| #22. Did you feel like you were treated roughly like pushed, beaten, slapped, pinched, physically restrained, or gagged? | |  |  |
|  | 0 No, never | 292 | 97.00 |
|  | 1 Yes, once | 2 | 0.70 |
|  | 2 Yes, a few times | 3 | 1.00 |
|  | 3 Yes, many time | 4 | 1.30 |
|  |  |  |  |
| #23. Did the medical staff at the facility ask you or your family for money other than the official cost? | |  |  |
|  | 0 No, never | 293 | 97.40 |
|  | 1 Yes, a few times | 6 | 2.00 |
|  | 2 Yes, most of the time | 1 | 0.30 |
|  | 3 Yes, all the time | 1 | 0.30 |

| PCMC variable | | Number | | Percent | |  |
| --- | --- | --- | --- | --- | --- | --- |
| #24. Do you think there was enough health staff in the facility to care for you? | |  | |  | |  |
|  | 0 No, never | | 24 | | 8.00 | |
|  | 1 Yes, a few times | | 9 | | 3.00 | |
|  | 2 Yes, most of the time | | 110 | | 36.50 | |
|  | 3 Yes, all the time | | 157 | | 52.20 | |
|  |  | |  | |  | |
| #25. Did you feel the medical staff at the facility took the best care of you? | |  | |  | |  |
|  | 0 No, never | | 5 | | 1.70 | |
|  | 1 Yes, a few times | | 10 | | 3.30 | |
|  | 2 Yes, most of the time | | 157 | | 52.20 | |
|  | 3 Yes, all the time | | 128 | | 42.50 | |
|  |  | |  | |  | |
| #26. Did you feel you could completely trust the medical staff at the facility with regards to your care? | |  | |  | |  |
|  | 0 No, never | | 1 | | 0.30 | |
|  | 1 Yes, a few times | | 4 | | 1.30 | |
|  | 2 Yes, most of the time | | 112 | | 37.20 | |
|  | 3 Yes, all the time | | 183 | | 60.80 | |
|  |  | |  | |  | |
| #27. Thinking about the labor and postnatal wards, did you feel the health facility was crowded? | |  | |  | |  |
|  | 0 No, never | | 131 | | 43.50 | |
|  | 1 Yes, once | | 102 | | 33.90 | |
|  | 2 Yes, a few times | | 58 | | 19.30 | |
|  | 3 Yes, many time | | 9 | | 3.00 | |
|  |  | |  | |  | |
| #28. Thinking about the wards, washrooms and the general environment of the health facility, will you say the facility was very clean, clean, dirty, or very dirty? | |  | |  | |  |
|  | 0 Very dirty | | 0 | | 0 | |
|  | 1 Dirty | | 21 | | 7.00 | |
|  | 2 Clean | | 270 | | 89.70 | |
|  | 3 Very clean | | 9 | | 3.00 | |

| PCMC variable | | Number | Percent |
| --- | --- | --- | --- |
| #29. Was there water in the facility? | |  |  |
|  | 0 No, never | 1 | 0.30 |
|  | 1 Yes, a few times | 1 | 0.30 |
|  | 2 Yes, most of the time | 23 | 7.60 |
|  | 3 Yes, all the time | 275 | 91.40 |
|  |  |  |  |
| #30. Was there electricity in the facility? | |  |  |
|  | 0 No, never | 0 | 0 |
|  | 1 Yes, a few times | 1 | 0.30 |
|  | 2 Yes, most of the time | 9 | 3.00 |
|  | 3 Yes, all the time | 290 | 96.30 |
|  |  |  |  |
| #31. In general, did you feel safe in the health facility? | |  |  |
|  | 0 No, never | 0 | 0 |
|  | 1 Yes, a few times | 3 | 1.00 |
|  | 2 Yes, most of the time | 24 | 8.00 |
|  | 3 Yes, all the time | 273 | 90.70 |
|  |  |  |  |
| *Excluded from final India scale: #15 (Support anxiety), #27 (Crowding), #29 (Water), #30 (Electricity) | | | |
| Excluded from final Kenya scale: #23 (Bribes) | |  |  |
