# Supplementary material for: Validation of the person-centered maternity care scale at governmental health facilities in Cambodia
Source: PLoS One. 2023 Jul 6;18(7):e0288051. doi: 10.1371/journal.pone.0288051 (PMC10325110; doi:10.1371/journal.pone.0288051)
Supplement: S4 Table — (DOCX) [file pone.0288051.s004.docx]

S4 Table. Exploratory factor analysis result of 29 items of the Kh-PCMC scale
